# Ultraviolet Radiation From a Plant Perspective: The Plant-Microorganism Context

**DOI:** 10.3389/fpls.2020.597642

**Published:** 2020-12-15

**Authors:** Lucas Vanhaelewyn, Dominique Van Der Straeten, Barbara De Coninck, Filip Vandenbussche

**Affiliations:** ^1^Laboratory of Functional Plant Biology, Department of Biology, Ghent University, Ghent, Belgium; ^2^Plant Health and Protection Laboratory, Division of Crop Biotechnics, Department of Biosystems, KU Leuven, Leuven, Belgium

**Keywords:** ultraviolet-B, ultraviolet-A, plants, plant-microorganism interaction, ultraviolet, UV-C

## Abstract

Ultraviolet (UV) radiation directly affects plants and microorganisms, but also alters the species-specific interactions between them. The distinct bands of UV radiation, UV-A, UV-B, and UV-C have different effects on plants and their associated microorganisms. While UV-A and UV-B mainly affect morphogenesis and phototropism, UV-B and UV-C strongly trigger secondary metabolite production. Short wave (<350 nm) UV radiation negatively affects plant pathogens in direct and indirect ways. Direct effects can be ascribed to DNA damage, protein polymerization, enzyme inactivation and increased cell membrane permeability. UV-C is the most energetic radiation and is thus more effective at lower doses to kill microorganisms, but by consequence also often causes plant damage. Indirect effects can be ascribed to UV-B specific pathways such as the UVR8-dependent upregulated defense responses in plants, UV-B and UV-C upregulated ROS accumulation, and secondary metabolite production such as phenolic compounds. In this review, we summarize the physiological and molecular effects of UV radiation on plants, microorganisms and their interactions. Considerations for the use of UV radiation to control microorganisms, pathogenic as well as non-pathogenic, are listed. Effects can be indirect by increasing specialized metabolites with plant pre-treatment, or by directly affecting microorganisms.

## Introduction

Life on Earth is exposed to the light spectrum ranging from ultraviolet-B (UV-B) to infrared wavelengths (295–2500 nm), hereafter referred to as natural radiation. The ultraviolet (UV) part of the electromagnetic spectrum comprises three classes: UV-C (200–280 nm), UV-B (280–315 nm) and UV-A (315–400 nm) with only UV-B and UV-A reaching the earth’s surface. Radiation with wavelengths below 290 nm declines to undetectable levels (Schäfer and Nagy, 2006). Although UV radiation is only a minor fraction of the sunlight reaching Earth’s surface, it causes significant biological effects on organisms which can affect plant-phyllosphere interactions, and indirectly plant-rhizosphere interactions, with most studies performed on UV-B (Robson et al., 2015; Carvalho and Castillo, 2018).

Land plants, fungi, and bacteria have several fundamental characteristics in common. While growing, they have minimal possibilities for rapid long-distance displacement and therefore they usually adapt their growth and metabolism to incoming light with functionally overlapping pigments. Plants and microorganisms most often occur in the same terrestrial ecosystems with an equivalent natural climatic environment. The plant phyllosphere gets exposed to UV radiation but the optical properties – as determined by the architecture – of plants, fungi and bacteria differ. Plants have structurally organized multilayer tissues throughout their life cycle, fungi usually have mono-layered mycelia (with fruiting bodies as multi-layered exceptions), and bacteria exhibit significantly less complex organization, as single cells or biofilms. Hence the effect and impact of UV radiation on these organisms will be fundamentally different, ranging from biomolecule damage to secondary metabolite production (Table 1). Interestingly, these UV induced effects are usually dependent on the radiation intensity and developmental stages (Moreira-Rodriguez et al., 2017). UV irradiation has the potency to redirect the carbon flux resulting in changes in several classes of primary and secondary metabolites (recently often referred to as specialized metabolites) such as carotenoids, phenolics, glucosinolates and even chlorophylls, with associated effects in the phyllosphere (Katerova et al., 2012; Mewis et al., 2012; Heinze et al., 2018; Vandenbussche et al., 2018). Some of the specialized metabolites produced by plants in response to UV relate with defense responses and interactions with phyllosphere components (Demkura and Ballare, 2012; Mewis et al., 2012). Moreover, specialized metabolite accumulation is often ascribed to hormonal changes in the plants, especially due to an increased expression of genes of the salicylic acid (SA) and jasmonate (JA) pathways, suggesting the stimulation of defense mechanisms that potentially impact microorganisms and other leaf dwellers (Vanhaelewyn et al., 2016a; Vandenbussche et al., 2018). Much like it is the case in other eukaryotes, light and other irradiation, including the UV range, have a very diverse impact on filamentous fungal development and metabolism (Tisch and Schmoll, 2010). The UV related changes in fungal development and metabolism are orchestrated by a set of specific photosignaling pathways.

**TABLE 1:** UV perception and responses across organisms.

	Plants	Fungi	Bacteria
**Photoreceptors**			
UV-A UV-B UV-C	CRYs, LOV UVR8, LOV PHOT1, (UVR8?)	CRYs, LOV, BLUF Yes, unknown ?	CRYs, LOV-HK, BLUF No ?
**Responses**	DNA repair↑ Secondary metabolites↑ Defense responses↑ Growth↓ Phototropism	DNA repair↑ Pigments↑ light-dependent development↑ circadian clock Cercosporin production↑ (a) sexual reproduction↑ or ↓ Phototropism Germination↓ Conidiation↑ or ↓	DNA repair↑ Secondary metabolites↑
**DNA repair**	CPD Photolyase 6-4 Photolyase	CRY1 BcRadl Bcin01g08960	CPD uvrA Rul AB plasmid
**Specialized metabolites**	Flavones, flavonols, isoflavonoids, anthocyanins, carotenoids, pinene, artemisinin, alkaloids glucosinolates	DHN melanin, DOPA-melanin, carotenoids	Pyocyanin, siderophores, carotenoids
**Defense hormones**	Jasmonate Salicylic acid	N.A.	N.A.

*Plants, fungi, and bacteria have similar and distinct UV responses, potentially influencing their internal relations. N.A., not applicable; ?, not reported. ↑: increases; ↓: decreases.*

Here we review how plants, fungi and bacteria perceive and respond to UV with focus on plants and plant-microbe interactions. An overview of their response mechanisms is presented, and the consequences for their growth and survival are discussed and summarized in Table 1. While we do contemplate on the potential of the application of UV in plant protection, we primarily provide a broad overview on the effects and possibilities of UV radiation. Despite the increasing evidence for the occurrence and potential role of Cyanobacteria in the phyllosphere (Rigonato et al., 2012; Singh et al., 2019), they will be kept out of consideration in this manuscript. This review provides a global overview and is a basis to guide scientists and growers to make educated decisions for UV treatments in various applications.

### Detection of Ultraviolet Radiation

**FIGURE 1:**
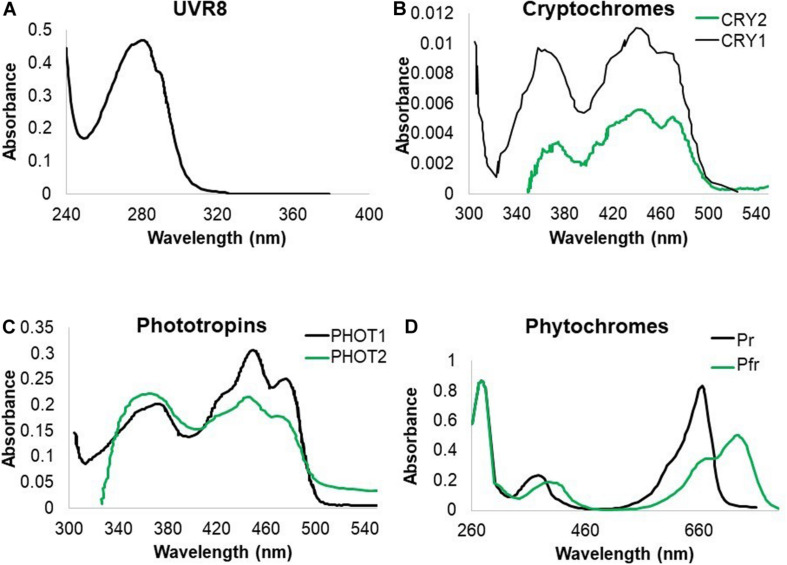
Absorption spectra of UV-B absorbing photoreceptors in *Arabidopsis*. **(A)** UVR8 absorption is recorded in the UV-C and UV-B region of the spectrum. **(B)** CRY1 and CRY2 absorb both strongly in blue and UV-A radiation with indication that CRY1 strongly absorbs in the UV-B band. **(C)** PHOT1 and PHOT2 both absorb mainly in the blue and UV-A wavelengths of the spectrum with indications that PHOT1 also absorbs UV-B. **(D)** PHY proteins have an absorption peak in red and far red wavelengths but also absorb UV-A and UV-B (Ahmad et al., 2002; Kasahara et al., 2002; Sang et al., 2005; Christie et al., 2012).

Living organisms developed photoreceptors in order to respond to light and optimize growth. Photoreceptors absorb specific wavelengths of radiation, triggering a cascade of events, leading to biological responses. Here we illustrate how plants and microorganisms perceive UV and summarize subsequent downstream responses. Plants have a wide set of photoreceptors, each of which has a specific absorption spectrum. Some of the known photoreceptors absorb in the ultraviolet region. UV responses are ascribed to the sensing by cryptochromes (CRYs) (Ahmad et al., 2002), phototropins (PHOTs) (Christie and Briggs, 2001; Vandenbussche et al., 2014), phytochrome A (PHYA) (Shinomura et al., 1996) and UVB-RESISTANCE 8 (UVR8) as a UV-B specific sensor (Heijde and Ulm, 2012). To date, no reports on a UV-C specific photoreceptor exist. Nevertheless, it is tempting to speculate that some of the known photoreceptors such as UVR8 and CRYs can be triggered by UV-C given their absorbance in that part of the spectrum (Figure 1), thus possibly playing a role in plant responses to UV-C (Table 1). The absorption spectrum due to chromophores in a protein context and the action spectrum of photoreceptors are very often correlated. Therefore, one can assume that photoreceptors will be triggered at any wavelength within the absorption range of the chromophore. Indeed, while phototropins are mostly regarded as blue light photoreceptors, they do display activity at wavelengths lower than 350 nm (Vandenbussche et al., 2014). Furthermore, homology of CRYs which function in the UV-A region with photolyases, is indicative for CRY activity in the UV region. It is however, noteworthy that the absorption and action spectrum for some photoreceptors are not documented below 320 nm (Figure 1) and therefore effects in the lower wavelengths, mediated by these photoreceptors cannot be excluded.

Comparable to plants, many fungi are exposed to natural radiation during their life cycle. Despite not being photoautotrophs, they have developed mechanisms to perceive and respond to radiation from the UV to the far-red part of the spectrum. Historically, *Phycomyces* photobiology has received a lot of attention, including its responses in the UV-range. While positive phototropism is reported for UV-A, *Phycomyces* bends away from UV-B and UV-C. These opposite responses are genetically independent (Martin-Rojas et al., 1995). Importantly, each type of UV can have major differences in responses given that UV-A activates vegetative spores of *Phycomyces* while they are killed by UV-C (Campuzano et al., 1996). Many studies on the effect of UV radiation on fungi reported direct killing of conidia. UV-A, on the other hand, promotes conidiation while sublethal doses of UV-A and UV-B reduce conidial germination (Braga et al., 2015). Additionally, UV radiation can affect hyphal growth and toxin production (Thind and Schilder, 2018).

Also bacterial survival and growth can be heavily affected by UV radiation but negative impacts rely on the diverse protection strategies bacteria developed to cope with UV-stress. UV sensitivity of bacteria is often linked with the habitat they are living in. Especially, the effect of UV radiation and UV tolerance mechanisms have been well studied in Cyanobacteria since these photosynthetic organisms cannot avoid solar energy (Rastogi et al., 2014). Finally, UV-C radiation, primarily at 245 nm, is well-known for its killing activity on microorganisms and is therefore often used for disinfection purposes (Urban et al., 2016).

#### UVR8 Serves as a UV-B Photoreceptor in Plants Under Natural Irradiation

Plant UVR8, with tryptophans acting as chromophores, absorbs in both the UV-B and UV-C part of the spectrum (Figure 1A; Christie et al., 2012). In recent years, the UVR8 pathway was well documented for the UV-B response and an array of physiological effects were ascribed to UVR8. Examples are: inhibition of hypocotyl elongation, induction of photoprotective pigment biosynthesis, increase of UV-B tolerance and augmented survival, strengthening of defense responses (Jenkins, 2017; Tossi et al., 2019), and a role in phototropism in hypocotyls and inflorescence stems (Vandenbussche et al., 2014; Vanhaelewyn et al., 2019). Upon UV-B exposure, inactive UVR8 dimers monomerize in the cytoplasm and accumulate in the nucleus where downstream signaling is induced (Kaiserli and Jenkins, 2007; Yin et al., 2016). Key components in this pathway include elongated hypocotyl 5 (HY5), HY5 homolog (HYH) and constitutively photomorphogenic 1 (COP1) (Ulm et al., 2004; Brown et al., 2005; Oravecz et al., 2006; Favory et al., 2009; Binkert et al., 2014; Yin et al., 2016). The current literature stipulates that UVR8 monomers avert the breakdown of HY5 by preventing the interaction between COP1 and HY5, leading to HY5 accumulation and signaling, accompanied with phytochrome interacting factor 5 (PIF5) and PIF4 destabilization and thus growth inhibition (Jenkins, 2017; Sharma et al., 2019; Tavridou et al., 2020). Moreover, this effect is enhanced by UVR8 binding to WRKY DNA-binding protein 36 (WRKY36), which usually acts as a HY5 transcriptional inhibitor (Liang et al., 2018). Importantly, HY5 induces flavonoid biosynthesis through upregulation of genes such as *chalcone synthase* (*CHS*) and *flavonol synthase* (*FLS1*) and *chalcone flavanone isomerase* (*CHI*). The UVR8 dependent upregulation of flavonoid biosynthesis, mainly serves to create a sunscreen and thus contributes to the protection of macromolecules against UV-B (Jenkins et al., 2001; Hollosy, 2002; Stracke et al., 2010a; Feher et al., 2011; Binkert et al., 2014). To date, despite the homology with the guanine nucleotide exchange factors regulator of chromatin condensation (RCC), no true UVR8 is reported in fungi and bacteria, while being conserved from green algae to higher plants in the plant kingdom (Binkert et al., 2016). Besides UVR8 mediated signaling, low fluence rate UV-B, as little as 0.1 μmol m^–2^s^–1^, can activate UVR8 independent gene expression of the transcription factor *Arabidopsis* NAC domain containing protein 13 (*ANAC13*), supporting the existence of a UVR8-independent pathway in UV-B signaling. Furthermore, this regulation is also independent of PHOT, CRY, phytochromes A, B, D, COP1, and HY5/HYH (O’Hara et al., 2019).

#### CRYs Act as Universal Blue and UV Photoreceptors With Similar Functions

Cryptochromes are pterin and flavin based blue light photoreceptors, well-documented in animals, plants, fungi and bacteria. Interestingly, in plants CRY1 and CRY2 both absorb in the UV-A range with CRY1 even absorbing UV-B wavelengths (Figure 1B). Therefore, it is generally accepted that cryptochromes mediate both blue and UV responses alike. Upon activation, CRY proteins are phosphorylated, form homodimers and undergo a conformational change, exposing the C-terminus, allowing physical interactions with signaling factors such as COP1 (Yang et al., 2000, 2001; Wang et al., 2001; Sang et al., 2005) and the COP1/SPA complex (Holtkotte et al., 2017). CRYs share sequence similarity with photolyases (see further), but are – with the exception of CRY-DASH (CRY3) – missing photolyase activity necessary to repair UV-induced DNA damage (Sancar and Sancar, 1987; Ahmad and Cashmore, 1993; Selby and Sancar, 2006). Similar to PHOTs and ZEITLUPE, a circadian photoreceptor (Kim et al., 2007), CRYs have a flavin chromophore. CRYs play an important role in photomorphogenic growth responses, regulating hypocotyl and cotyledon growth, and controlling specialized metabolite production such as anthocyanins (Liu et al., 2018). CRY1 was shown to be important for defense, stress and detoxification responses. For example, in *Arabidopsis*, CRY1 promotes R protein-mediated plant resistance to *Pseudomonas syringae* (Wu and Yang, 2010).

Cryptochromes appear universal photoreceptors, present in most, if not all cellular organisms and in fungi they constitute a large class with combined or unique signaling and photolyase function [Reviewed in Cohrs and Schumacher (2017)]. In some fungi light also regulates pigment accumulation, as reported in the phytopathogenic *Botrytis cinerea* and in *Fusarium fujikuroi* pigment accumulation is known to be CRY dependent (Veluchamy and Rollins, 2008; Castrillo et al., 2013; Schumacher, 2016). Specific plant-microbe interactions as well as fungal hosts mentioned throughout the review are indicated in Supplementary Table 1. CRYs can steer light-dependent development of *Sclerotinia sclerotiorum* (sclerotial and apothecial development) and *F. fujikuroi* (formation of macroconidia). Moreover, CRY-DASH in *Neurospora crassa* influences light-regulated transcription and is necessary for phase entrainment of the circadian clock (Froehlich et al., 2010; Nsa et al., 2015). Some fungal CRYs belonging to the CRY-DASH family, using 5,10-methenyltetrahydrofolate (MTHF) and flavin adenine dinucleotide (FAD) as chromophores, have DNA repair activity as a photolyase (Tagua et al., 2015). In addition, the pyrimidine (6-4) pyrimidone dimers (6-4PPs) photolyase of *Cercospora zeae-maydis* is a regulator of cercosporin biosynthesis and represses the asexual reproduction by spore formation (Bluhm and Dunkle, 2008) while *Aspergillus nidulans* cyclobutane pyrimidine dimers (CPD) photolyase represses sexual development (Bayram et al., 2008). The CRY-DASH of *Trichoderma atroviride* has no clear regulatory function (Garcia-Esquivel et al., 2016) but its CPD photolyase negatively regulates its own photoinduction (Berrocal et al., 2008). Besides the cry-mediated DNA repair activity, some fungi contain true photolyases, while harboring DNA binding CRYs which are incapable of repairing damage (Froehlich et al., 2010). Thus, in fungi, CRYs and photolyases (including both CPD and 6-4 photoadduct photolyases) are not necessarily functionally separated, and one enzyme sometimes accounts for both photoreactivation and photomorphogenic functions. In *Botrytis cinerea*, blue light represses conidiation while near UV (300–400 nm) induces conidiation. However, the mechanism behind this induction remains elusive and seems to be independent from *B. cinerea* CRY receptors. In contrast, BcCRY2 even acts as a repressor of conidiation (Cohrs and Schumacher, 2017). Therefore, it has been hypothesized that *B. cinerea* contains a yet unknown photoreceptor that perceives UV-light (Cohrs and Schumacher, 2017).

Bacteria also hold several flavin based photoreceptors. To date, such proteins have been mainly classified as blue light receptors, even though their absorption spectra often show peaks into shorter wavelengths, indicating possible activation by UV-A. Some bacteria have CRY-DASH proteins, which have MTHF as antenna pigment for UV, transferring energy to FAD (Saxena et al., 2005; Moon et al., 2012). CRY activation in bacteria can affect motility and attachment, while blue light sensing using FAD (BLUF) proteins were found to control motility, adhesion, and production of exopolysaccharides and biosurfactants (Kraiselburd et al., 2017).

#### Light-, Oxygen, or Voltage Domains (LOV Domains) Are Universal UV-A and UV-B Photoreceptors

Phototropins were extensively investigated in relation with blue light phototropic responses but also absorb in the UV-A and UV-B region (Okajima et al., 2011; Figure 1C) consistent with the action spectra (Ahmad et al., 2002; Kasahara et al., 2002; Vandenbussche et al., 2014). Moreover, PHOT1 function was shown to be responsible for rapid decrease in seedling growth rate under UV-C conditions (Mageroy et al., 2010). PHOTs are membrane-associated photoreceptors which trigger a downstream signal transduction cascade (Hohm et al., 2013). *Arabidopsis* has two PHOTs, namely PHOT1 and PHOT2. These two PHOTs are very similar in structure, amino acid sequence and domain organization (Briggs et al., 2001; Sakai et al., 2001). PHOTs consist of an N-terminal photosensory domain and a C-terminal Ser/Thr protein kinase domain for signal output. The photosensory domain consists of two LOV domains, namely LOV1 and LOV2, together referred to as LOV. Both LOV1 and LOV2 comprise 110 amino acids (Christie et al., 1998). Yet, their functions are different, LOV1 regulates receptor di/multimerization (Salomon et al., 2004) while LOV2 regulates the C-terminal Ser/Thr kinase domain (Christie et al., 2002). PHOTs have a flavin mononucleotide (FMN) chromophore which in this case binds both LOV1 and LOV2 (Christie et al., 1999). FMN is non-covalently associated with LOV in darkness, while in light conditions, FMN gets activated and binds LOV covalently through a conserved cysteine residue (Christie et al., 1999; Christie, 2007). Under the condition of moderate or high light, PHOT1 and PHOT2 conjoin to spark downstream signaling, whereas under low light conditions, PHOT1 acts as the plant’s exclusive directional blue light and UV-A photoreceptor (Sakai et al., 2001). Recent work indicates that UV-B can activate PHOTs in a similar way as described under blue light (Vanhaelewyn et al., 2016b). The strength of the responses correlate with light intensity, as demonstrated for stomatal opening, leaf blade expansion and positioning of the chloroplasts (Sakai et al., 2001; Han et al., 2013).

Also in fungi, blue/UV-A photoreceptors that contain a LOV domain have been characterized. Most prominent is the White Collar Complex (WCC), a complex formed by WC1 and WC2 and named after the original mutation found in *N. crassa* with white mycelium and pigmented conidia. In *N. crassa*, the LOV domain of WC-1 binds FAD, allowing photoperception (Froehlich et al., 2002; He et al., 2002). As this protein contains a Zn finger DNA binding domain, extensive signaling routes appear unnecessary. Nevertheless, WC-1 interacts with WC-2, a non-chromophore containing Zn finger protein, to form WCC that binds promoters of light-inducible genes (He and Liu, 2005). WC-like proteins are described for multiple fungi (Lu et al., 2005; Terashima et al., 2005; Idnurm et al., 2006; Silva et al., 2006), indicating a light perception mechanism that may be conserved in a wide variety of fungi. In *Phycomyces*, the WC-1 homolog MADA (Max Delbrück A) is responsible for positive phototropism in blue light and UV-A (Campuzano et al., 1994). Mechanistically, MADA interacts with the WC-2 homolog MADB to promote the direction of growth of sporangiophores but also to regulate gene transcription in mycelia (Sanz et al., 2009). Additionally, other MAD type proteins can participate in the WCC. In *T. atroviride*, blue light receptors (BLRs), homologs of WC1 and WC2, are required for photoinduction of conidiation and gene expression regulated by blue light (Schmoll et al., 2010). Apart from WC proteins, other small LOV domain proteins in fungi include VIVID (VVD) and its homolog ENVOY1 (ENV1), regulators of WCC (Fuller et al., 2015).

In general, many plant-associated bacteria have blue light receptors. Plant pathogenic *Ralstonia*, *Pseudomonas* and *Xanthomonas* have LOV and/or BLUF proteins (Mandalari et al., 2013; Ricci et al., 2015; Kraiselburd et al., 2017). Although the effects of these proteins have been mainly studied in blue light, because of their absorbance properties (Cao et al., 2008), it is likely that UV-A has a similar mode of action. In *Xanthomonas citri*, the role of LOV proteins is related to colonizing the host plant, including exopolysaccharides, adhesin and flagellum synthesis and consequent motility, adhesion, biofilm formation and oxidative stress resistance (Kraiselburd et al., 2017). In *Pseudomonas syringae*, the role of LOV proteins appears diverse. In *P. syringae* pathovar tomato, LOV domain proteins regulate the switch to a non-motile lifestyle and growth inhibition, while in *P. syringae* pv *syringae* they are supposed to promote swarming motility (Wu et al., 2013; Kraiselburd et al., 2017).

#### Phytochromes Potentially Act as Minor UV Photoreceptors

Phytochromes sense red light with a bilin chromophore (Sharrock, 2008). The chromophore occurs in two interchangeable conformations, namely Pr and Pfr which have different spectral properties with major differences in the red region of the spectrum (Figure 1D). Pr absorbs red, and changes conformation, thereby shifting its absorption maximum into the far-red, leading to the Pfr form. This process is reversible upon absorbing far-red light. Besides red light detection, phytochromes also absorb in the UV-A and UV-B regions (Figure 1D) but there is limited evidence on UV induced phenotypes. Examples are UV-A-dependent chloroplast gene transcription in green leaves (Chun et al., 2001) and a role of phytochromes in UV-A induced anthocyanin biosynthesis (Oelmuller and Mohr, 1985). There are, however, no reports on PHY dependent antimicrobial responses which are provoked by UV radiation but it is tempting to speculate for a role for phytochromes in plant-microbe interactions, given that HY5 is somehow PHY regulated (van Gelderen et al., 2018), but given the lack of evidence, phytochromes will not be further elaborated here.

Also, for microorganisms, phytochromes do not seem to play an important role in UV detection and responses. Fungal phytochromes were initially discovered in the two model fungi *N. crassa* and *A. nidulans* with biliverdin as most likely natural chromophore and are now considered present throughout the Fungi kingdom (Blumenstein et al., 2005; Brandt et al., 2008). In bacteria, phytochromes are referred to as bacteriophytochromes. However, no clear UV responses are reported for these photoreceptors.

## Dna Damage

The exposure or application of organisms to UV light comes with risks. Besides active sensing and signal induction to UV radiation by photoreceptors, some UV induced morphological responses can be ascribed to the interaction of the high energetic UV radiation with molecules. In the UV spectrum, UV-A irradiation of plant tissues has been shown to have little DNA damaging effects because UV-A is not absorbed by DNA (Supplementary Figure 1a). However, DNA damage can still be indirectly caused by ROS generation issuing from photosensitizing reactions, especially by highly reactive singlet oxygen (^1^O_2_) (Alscher et al., 1997). On the other hand, higher energy UV irradiation such as UV-B and UV-C are strongly absorbed by nuclear, mitochondrial and chloroplast DNA (Supplementary Figure 1a), causing similar mutations.

### UV Induces Several Types of DNA Damage

Ultraviolet-B is very potent to cause chemical modifications and DNA damage in natural conditions (Sinha and Hader, 2002; Douki et al., 2003; Rastogi et al., 2010). UV-B radiation generates several distinct alterations in DNA, named photoproducts. The most likely photoproducts inducing mutations are two different lesions that unite adjacent pyrimidine residues in the same strand. The major lesions are CPDs, accounting for about 75% of UV-B mediated DNA damage (Takahashi et al., 2011), and 6-4PPs. Minor DNA damage include hydrated bases, oxidized bases and single-strand breaks (Ballare et al., 2001; Takahashi et al., 2011). Interestingly, CPDs and especially 6-4PPs cause DNA bending, with unwinding reported for 6-4PPs, causing growth retardation, lethality or mutagenesis (Tuteja et al., 2009). These types of DNA damage are usually not observed under low UV-B rates (<1 μmol m^–2^s^–1^), which are sufficient to stimulate photomorphogenic and protective responses (Kim et al., 1998; Frohnmeyer et al., 1999). Given the unstable nature of DNA and the high occurrence of CPDs, plants developed sophisticated systems to deal with this type of DNA damage.

### Plants Have a Plethora of DNA Repair Possibilities

The major pathway for UV-B induced repair is the photorepair or photoreactivation. By absorbing blue/UV-A radiation, CPD photolyases and 6-4PP photolyases can monomerize the UV-B induced dimers. These photolyases specifically bind to DNA lesions and remove the CPDs and 6-4PPs (Sancar, 2003; Bray and West, 2005). Interestingly, UV-B sensitive rice cultivars were shown less able to repair CPDs as compared to resistant cultivars, while over-expression of CPD photolyase resulted in significantly higher tolerance to UV-B damage (Teranishi et al., 2012). Not surprisingly, the expression of the CPD photolyase gene (*PHR*) is predominantly controlled by UV-B in a UVR8 dependent manner, but also by UV-A and blue light in a CRY-dependent way (Li et al., 2015). In addition to the photoreactivation mechanism, there is a light-independent repair or dark repair mechanism. This mainly includes nucleotide excision repair (NER), mismatch repair (MMR), and base excision repair (BER). NER mainly recognizes conformational changes in DNA, rather than specific types of DNA damage (Boubriak et al., 2008) and is able to repair CPDs and 6-4PPs (Tuteja et al., 2001). Double strand break repair and homologous recombination, extensively reviewed before (Gill et al., 2015), are not often observed after UV damage.

### Fungi Have Their Own DNA Repair Mechanisms

Among plant pathogens, the photodamage responses in *B. cinerea* have received quite some attention (Table 1). Like other fungi, *B. cinerea* has a functional WCC (Canessa et al., 2013). Furthermore it contains a separate functional photolyase (CRY1) and a CRY-DASH (CRY2) that does not appear to participate in photoreactivation (Cohrs and Schumacher, 2017). Apart from the afore mentioned enzymes, and true photolyases like phr in *N. crassa* (Shimura et al., 1999) and *T. atroviride* (Berrocal-Tito et al., 2007), fungi also contain other DNA repair mechanisms (Goldman et al., 2002; Inoue, 2011). The respective genes are often called MUS, for mutagen sensitive, or UVS, for UV sensitive. *Aspergillus nidulans UvsI* is an ortholog of Rev3 in yeast, a polymerase that duplicates past UV-induced lesions, thus generating mutations (Han et al., 1998). Of particular interest is the DNA repair mechanism in *N. crassa*, wherein two nucleotide excision repair pathways were found (Inoue, 2011). The first is controlled by mus-38 endonuclease (homolog of yeast Rad1 and UVH1 of Arabidopsis thaliana ), the other by mus-18, a unique endonuclease capable of removing UV generated photoproducts (Yajima et al., 1995). These endonucleases show some functional redundancy (Hatakeyama et al., 1998). Because of the conservation of nucleotide excision repair systems in eukaryotes, it is likely that this is also the case within the fungi. In this respect, *B. cinerea* has a MUS-38 (BcRad1) ortholog, and a MUS-18 ortholog (Bcin01g08960).

### Bacteria Can Also Survive UV Radiation

Comparable to plants and fungi, bacteria also possess photoreactivation properties (Kelner, 1949), which rely on rapid and efficient light dependent DNA repair by photolyase activity (Sancar, 1996). These photolyases can also be activated by blue light, as well as UV-A, owing to their pterin and flavin chromophore. Apart from photolyase activity, bacteria, including plant pathogens, have nucleotide excision repair which functions in the absence of light (Kim and Sundin, 2001; Gunasekera and Sundin, 2006; Table 1). Not surprisingly, phyllosphere bacteria and especially Cyanobacteria, which are exposed to solar radiation under natural conditions, show a degree of tolerance to UV-B. To this end they produce an array of enzymatic (e.g., catalase, superoxide dismutases, glutathione and ascorbate peroxidases) and non-enzymatic (carotenoids, α-tocopherols, ascorbic acid, and glutathione) antioxidants to counteract the production of ROS, an extracellular polysaccharide layer to avoid UV damage as well as the production UV-B absorbing/screening compounds such as mycosporine-like amino acids (MAAs) and scytonemin (Rastogi et al., 2014). Furthermore, *Pseudomonas* sp. have an additional method for DNA repair called mutagenic DNA repair, which helps in the protection against UV-B (Sundin and Murillo, 1999). The rulAB plasmid operon conferring this characteristic is of importance for UV-tolerance for the maintenance of the bacterial population during infection, and provides epiphytic fitness (Kim and Sundin, 2000; Cazorla et al., 2008). It is not known if DNA repair is under the control of a photosignaling pathway in bacteria.

### Not All UV Inflicted Damage Can Be Repaired

Given the strong genotoxic nature of UV-C radiation and its detrimental effects on all types of microorganisms, including fungi, bacteria, and their spores, it is frequently used for its germicidal potential. In general, the most resistant structures are fungal and bacterial spores, which need large doses of UV-C to be inactivated, while non-sporulating bacteria are more sensitive (Gayan et al., 2013). Indeed, 254 nm radiation effectively and rapidly kills a variety of bacteria (Wright et al., 2000; Ozcelik, 2007; Jones et al., 2014), but also UV-B was shown useful to kill off bacteria (Oppezzo, 2012).

## UV Regulated Specialized Metabolites and Hormones, Leading to Augmented Plant-Microbe Interactions

Specialized metabolites play a major role in plant-environment interactions by acting as antioxidants, signaling molecules, protective compounds against abiotic stress and pathogens, herbivore feeding deterrents and even pollinator attractants. To cope with UV-induced photodamage, organisms developed an arsenal of photoprotective molecules such as melanins (humans and animals), flavonoids (plants), mycosporines (fungi) and mycosporine-like amino acids (MAAs, Cyanobacteria, algae, and animals) (Sinha et al., 2007). The accumulation of specialized metabolites is particularly well studied upon UV-B radiation. Plant specialized metabolites are divided in three chemically distinct groups: phenolics, terpenes and nitrogen-containing compounds. The most relevant specialized metabolites that affect plant-microbe interactions in a UV context are listed below.

### Phenolics Are Aromatic Benzene Ring Compounds With One or More Hydroxyl Groups Produced by Plants Mainly for Protection Against Stress

The phenolics group consists of nearly 10,000 individual compounds with a large chemical and biological diversification. Flavonoids are one of the largest classes of plant phenolics. The basic carbon skeleton consists of 15 carbons arranged in two aromatic rings, connected by a three-carbon bridge. Flavonoids are classified in different groups, mainly based on the degree of oxidation of the three-carbon bridge. Here we focus on flavones, flavonols isoflavonoids and anthocyanins. Flavones and flavonols are present in flowers and leaves of all green plants and generally absorb radiation of shorter wavelengths, with peaks in the 240–280 nm and 315–400 nm range (Supplementary Figure 1b; Cerovic et al., 2002). Besides a sun-screen, they can also perform antioxidant functions such as ROS scavenging near the site of generation, this is especially true for flavonoids with multiple hydroxyl groups (Gould et al., 2002; Schmitz-Hoerner and Weissenbock, 2003; Kytridis and Manetas, 2006). Upon UV-B exposure, flavonoids accumulate in the epidermal cells, leaf hairs and leaf wax (Harborne and Williams, 2000), filtering harmful UV-B rays while still ensuring passage of photosynthetically active radiation (PAR) light. Quercetin and kaempferol derivates accumulate in *Arabidopsis* seedlings (Stracke et al., 2010b) acting both as UV protectants and free-radical scavengers (Agati and Tattini, 2010). UV-B mediated flavonoid accumulation is UVR8 dependent (Demkura and Ballare, 2012; Morales et al., 2013), leading to an induction of CHS, chalcone flavanone isomerase (CHI), flavanone 3-hydroxylase (F3H) and flavonol synthase (FLS) (Hideg et al., 2013), all of which are HY5 inducible (Shin et al., 2013; Wu et al., 2019), Interestingly dihydroflavonol reductase (DFR) was only shown to be induced in UV-A conditions (Morales et al., 2013). However, the role of UVR8 in the induction of DFR transcript is not COP1 regulated under UV-B conditions (Oravecz et al., 2006) and therefore hints at an interaction between photoreceptors (Morales et al., 2013) below 350 nm (Morales et al., 2013; Rai et al., 2020). Not surprisingly, *Arabidopsis* plants missing CHS due to point mutations are much more sensitive to the effects of UV-B (Li et al., 1993).

Besides the sunscreen and antioxidant function to protect underlying photosynthetic tissues from damage, several types of flavonoids display antifungal, antibacterial and antiviral activities (van der Watt and Pretorius, 2001; Cushnie and Lamb, 2005). Generally, flavonoid accumulation leads to increased resistance against pathogens including, e.g., rust disease in cedar-apple (Lu et al., 2017) and powdery mildew in cucumber (Fofana et al., 2002; McNally et al., 2003). UV-B inducible flavones such as luteolin and apigenin have antimicrobial activity against a wide range of microorganisms and are especially known to inhibit the growth of food-borne pathogens (Cushnie and Lamb, 2005; Clayton et al., 2018). But also flavonols such as rutin are increased by UV-B treatment in *Nicotiana attenuata* and *Nicotiana longiflora* (Izaguirre et al., 2007; López-Gresa et al., 2011). They display antimicrobial activity (Pereira et al., 2007), and are induced upon *P. syringae* infection in tomato plants. Because of this widespread characteristic of flavonoids to inhibit spore germination of plant pathogens, synthetic flavonols, such as a derivative of quercetin have received extensive attention for medicinal fungal treatments (Harborne and Williams, 2000). Also chalcones such as phloretin and naringenin have been reported to display antimicrobial activities (Rauha et al., 2000; Koru et al., 2007; Vargas et al., 2013) with affected mobility of *Pseudomonas syringae pv. tomato* (Vargas et al., 2013). Further down the flavonoid pathway, anthocyanins which are usually associated with intense colors to attract insects, can also display antimicrobial effects, as they can destroy the cell wall of bacteria such as in *Salmonella* and *Escherichia coli* (Ma et al., 2019) and reduce fungal growth of e.g., *Geotrichum candidum* and *Candida albicans* (Wen et al., 2016). Similar observations were reported for leucoanthocyanidins such as leucocyanidin (Cushnie and Lamb, 2005). Furthermore, pro-anthocyanidins such as tannins (oligomeric flavonoids, as in wine) were also shown to display antimicrobial activity (Mayer et al., 2008) and against enterotoxigenic *E. coli* (Tang et al., 2017). Noteworthy, isoflavonoids are known for their phytoalexin function. The latter are antimicrobial compounds synthesized in response to fungal or bacterial infections, as well as upon pest attack. Isoflavonoids are derived from the flavonoid biosynthesis pathway via liquiritigenin or naringenin and consist of a broad structurally similar group of molecules such as isoflavones, isoflavonones, isoflavans. The isoflavonoid maackiain can inhibit the germination of plant pathogen spores (Harborne and Williams, 2000) and relates to *B. cinerea* resistance (Stevenson and Haware, 1999).

Besides flavonoids, sinapates also accumulate at ecologically meaningful doses of UV-B. Sinapates are building blocks for lignin and can increase resistance of *Arabidopsis* to *B. cinerea* in a UVR8 dependent way (Demkura and Ballare, 2012). Interestingly, biotic stress elicits MAMP triggered immunity (MTI), which in turn inhibits the previously described flavonoid accumulation under UV-B conditions due to a biosynthetic shift of phenylalanine based molecules from flavonoids to lignin and the phytoalexin scopoletin, serving as a physical defense barrier against pathogens (Schenke et al., 2019). The flavonoid downregulation is mainly due to the downregulation of the CHS gene with prevention of histone acetylation (Zhou et al., 2017).

**FIGURE 2:**
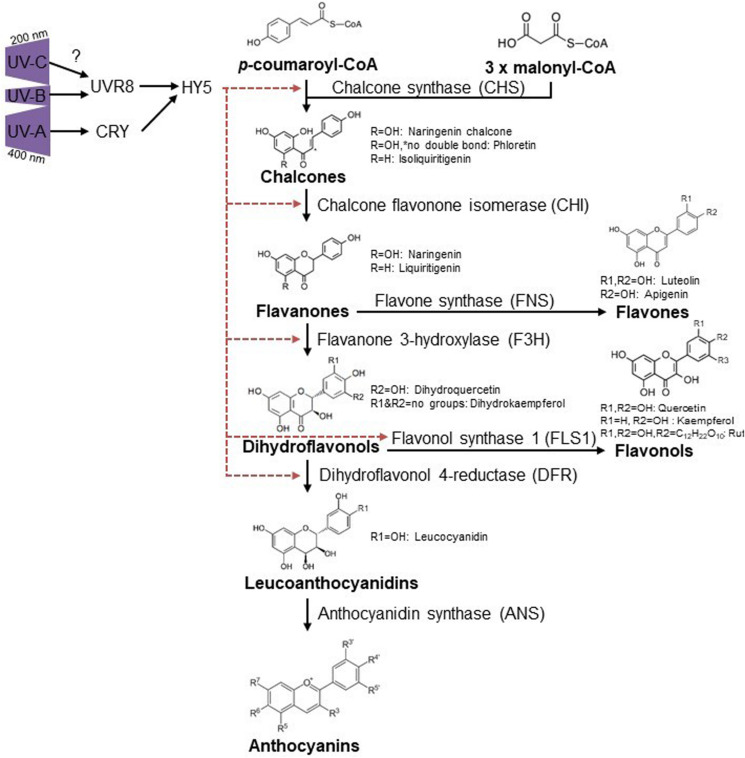
Ultraviolet induced flavonoid accumulation is UVR8 and CRY dependent. UV-B activates UVR8 (Kaiserli and Jenkins, 2007; Yin et al., 2016) which leads to HY5 accumulation and thus diverse flavonoid biosynthesis via CHS, CHI, FH3, and FLS1 (Hideg et al., 2013). CRYs are activated via UV-A and regulate HY5, causing flavonoid accumulation in a similar way as described for UVR8 but also DFR regulation was reported for UV-A conditions (Morales et al., 2013). R-groups are indicated for substances mentioned throughout the text. Red dashed arrows indicate induction of specific enzymes by HY5.

Flavonoid induction by UV-A is likely mainly induced through the CRY1 photoreceptor, providing that functional CRY1 is essential for chalcone synthase (CHS) expression (Fuglevand et al., 1996), with no roles described for PHOTs and UVR8 in this waveband (Verdaguer et al., 2017). Given that the absorption spectrum of UVR8 reaches into the UV-C spectrum and overlaps with the action spectrum for anthocyanin accumulation (Jiang et al., 2012), it is tempting to speculate that UV-C triggers the same pathway as in UV-B conditions (Figure 2).

### Terpenes Are Very Volatile and Constitute the Largest Class of Specialized Metabolites

Terpenes are generally synthesized from acetyl-CoA. Importantly, some terpenes can be considered as primary metabolites since they have well defined roles in plants, for example gibberellins (diterpenes), brassinosteroids and abscisic acid, some of which are discussed below. Carotenoids are tetraterpenes that function as accessory pigments during photosynthesis and protect against photooxidation. Carotenoids are strongly induced upon UV exposure. Several plant induced carotenoids were ascribed potent anti-*Helicobacter pylori* activity, namely violaxanthin, luteoxanthin, neoxanthin, antheraxanthin, and lutein (Molnar et al., 2010). Some terpenes can act as a repellent for insects and animals (Isman, 2006; Michaelakis et al., 2009; Tholl, 2015). Recently, terpenes are receiving more attention in the Cannabis industry for their “entourage effect” for medical and olfactory properties. Monoterpene synthase (QH6) is induced by HY5 (Gangappa and Botto, 2016), QH6 converts geranyl diphosphate to β-pinene and α-pinene. Pinene is one of the several terpenes which receives major attention for its antimicrobial and antioxidant functions, and which is reported as UV-B inducible (Salehi et al., 2019). Moreover, β-caryophyllene can protect plants against fungal and bacterial pathogens (Sharma et al., 2016; Nuutinen, 2018). Antimicrobial effects of terpenes in essential oils were also reported for camphene, caryophyllene, eucalyptol, humulene, pineneterpinolene *trans-*nerolidol, eugenol, terpineol, carveol, citronellol, and geraniol (Guimaraes et al., 2019). Many of these terpenes absorb in the UV-range and some terpenes are reported as UV-B inducible. One example is the sesquiterpene (E,E)-α-farnesene, which is also increased after jasmonic acid treatment (Liu et al., 2017). Another industrially important compound is artemisinin, a UV-B and UV-C inducible sesquiterpene (Rai et al., 2011), with non-enzymatic conversions in the terpenoid metabolism (Czechowski et al., 2016). Artemisinin is well-characterized for its anti-malarial effects while its derivates are also ascribed antifungal (Galal et al., 2005) and antibacterial functions (Appalasamy et al., 2014).

Associated with their terrestrial life, many fungi have acquired the capability of synthesizing compounds that cause pigmentation. Fungi produce carotenoids and 1,8-DHN-melanin-type or DOPA-melanin (Goncalves et al., 2012). Photo-induced melanization, presumably depending on the WCC complex, has been observed for several fungi (Fuller et al., 2015). Since melanins absorb in a very broad range of wavelengths, including UV, they protect fungi and their spores from UV radiation. A melanin mutant of *Cochliobolus heterostrophus*, a pathogen of maize, was for example unable to survive in the field (Keller, 2019). In plant pathogenic *Colletotrichum* species, *Venturia inaequalis*, *Magnaporthe grisea*, and *Diplocarpon rosae*, melanin type pigments are required for efficient host invasion. Their appressoria need melanin to sustain turgor pressure in order to effectively penetrate the host leaves (Kubo and Furusawa, 1991; Steiner and Oerke, 2007; Gachomo et al., 2010). Interestingly, fungi also accumulate carotenoids for UV protection. Carotenoid accumulation in *Neurospora* strains is related to latitude and UV radiation, with higher levels at higher latitudes, correlated with increased UV. Moreover in this organism, carotenoid accumulation is associated with UV-resistance (Luque et al., 2012). For several fungi it has been reported that production of carotenoids is induced in a WC-1 dependent manner (Fuller et al., 2015).

Many plant phyllosphere bacteria, e.g., *Clavibacter michiganensis*, *Curtobacteria*, and *Methylobacteria*, are known to be chromogenic and UV tolerant (Sundin and Jacobs, 1999; Yoshida et al., 2017). In *Methylobacteria*, the UV-absorbing molecule is similar to avobenzone, a compound used in commercial sun-blockers (Yoshida et al., 2017). Although no photoprotecting UV-absorbing pigment has been described for *P. syringae*, a member of the same genus, *Pseudomonas aeruginosa* can accumulate pyocyanin, which has strong absorption in short wave UV-B and UV-C. Furthermore, many *P. syringae* strains fluoresce green and yellow under UV radiation, due to accumulation of siderophores that usually absorb in the UV-B and UV-A range (Cody and Gross, 1987; Hirano and Upper, 2000; Bultreys et al., 2001; Rombouts et al., 2016). On well-watered tea plants, *Xanthomonas* sp. are affected by UV-B, while this was not the case for *Corynebacterium aquaticum* (Gunasekera and Paul, 2007). As the presence of UV screening compounds and UV-tolerance is species specific, it is no surprise that UV can affect the bacterial diversity in the phyllosphere (Kadivar and Stapleton, 2003). Some bacteria, including Flavobacterium and many Mycobacteria and Myxococci, are capable of accumulating photoprotective pigments, including carotenoids such as phytoene and phytofluene, which absorb in blue, UV-A and UV-B (Batra et al., 1971). While a pterin like molecule was suggested in the activation of carotenoid biosynthesis in UV-A in Myxococcus, the action spectra for the induction of carotenoids very often points to flavin dependence, with high responses in the UV-A and blue, but also in the UV-B range (Batra et al., 1971). Common to various families of non-phototrophic bacteria, carotenoid biosynthesis is under the control of a light-releasable repressor complex, LitR-AdoB12, based on a MerR (Mercury Resistance) transcriptional regulator and adenosyl B_12_, yet a photoperception pathway to activation of carotenoid biosynthesis has not been documented (Takano, 2016).

### Nitrogen-Containing Compounds Such as Alkaloids and Glucosinolates Are Well-Known Antiherbivore Defense Metabolites

Alkaloids and glucosinolates can be toxic to humans while others are shown valuable as medicine. Most of these metabolites are synthesized from common amino acids. The primary plant benefit of UV-B dependent upregulation of alkaloids may be related to their ability to quench singlet oxygen and thus confer protection against this toxic photosynthetic by-product (Larson and Marley, 1984). Alkaloids are generally water insoluble organic heterocyclic nitrogen compounds, some of which display antimicrobial functions (Cushnie et al., 2014). Most of the roles attributed to alkaloids are related to self-preservation of the organism and competitor inhibition. In plants, alkaloids can act as phytoanticipins and phytoalexins (plant antibiotics), thus protecting plants against infection (Gonzalez-Lamothe et al., 2009). Besides their natural function, alkaloids have inspired the development of several antibacterial drugs such as quinolones, metronidazole and bedaquiline (Bogatcheva et al., 2011; Hraiech et al., 2012; Parhi et al., 2012; Cushnie et al., 2014). Interestingly, UV-B was shown to induce certain plant alkaloids such as catharanthine (Ramani and Chelliah, 2007), lochnericine, serpentine, and ajmalicine up to 60% (Binder et al., 2009). Everything considered, it is tempting to speculate that UV treatment of plants may affect the plant-microbe interaction by inducing antimicrobial alkaloids.

Glucosinolates (GS) or mustard oil glycosides break down to release defensive substances. GS are generally found in the Brassicaceae and related families. GS are located within the vacuoles and are physically separated from β-thioglucosidase enzymes (myrosinases). Upon disruption of the plant tissue, myrosinases and GS get in contact and hydrolysis occurs, generating sulfate, glucose and an aglycone moiety. The aglycone moiety is unstable and is converted in either thiocyanates (TCs), nitriles and isothiocyanates (ITCs) (Delaquis and Mazza, 1995; Mithen, 2001; Vig et al., 2009). GS and their enzymatic hydrolysis products have been ascribed several biological activities including plant defense, especially ITCs are inhibitors of microbial activity, mainly documented in food preservation contexts (Chew, 1988; Gyawali and Ibrahim, 2014). Adverse effects were reported on insects, bacteria and fungi (Mewis et al., 2005, 2006; Halkier and Gershenzon, 2006; Saladino et al., 2016). In broccoli sprouts, UV-B irradiation affects the specialized metabolite profile in a similar way as observed during biotic stress. UV-B specifically induces glucosinolates (GS) such as 4-methoxy-indol-3-ylmethyl GS and 4-methylsulfinylbutyl GS (Mewis et al., 2012). GS hydrolysis products such as ITCs also have antibacterial effects to plant pathogenic bacteria such as *Agrobacterium tumefaciens*, *Pseudomonas tomato*, *Pseudomonas cichorii*, *Erwinia chrysanthemi*, *Xanthomonas juglandis* and *Xanthomonas campestris* (Aires et al., 2009). The modification of GS content is thus an interesting alternative to pesticide usage (Saladino et al., 2016).

In response to UV, fungi and Cyanobacteria produce mycosporine and mycosporine-like amino acids (MAAs), colorless, water-soluble compounds composed of a cycloheximine or cyclohexenone chromophore which is conjugated with a nitrogen or imino alcohol substituent (Sinha et al., 2007). Mycosporines and MAAs are UV-absorbing pigments and have a role as “sunscreen compounds” (Oren and Gunde-Cimerman, 2007) and potentially may serve as antioxidant molecules. Besides protection to UV, there is no information available whether these molecules affect plant-microbe interactions. Also fungal mycotoxins can be affected by UV radiation and have been associated with UV tolerance including, e.g., aflatoxin and citrinin produced by *Aspergillus flavus* and *Penicillium verrucosum*, respectively (Braga et al., 2015).

### UV-Augmented Plant Hormones

Finally, besides the defense-related role of some UV induced specialized metabolites, there are numerous reports on the role of plant hormones against biotic stress. UV-B was studied extensively in relation to all plant hormones and was recently reviewed in Vanhaelewyn et al. (2016a). The two main plant hormones for defense responses against microorganisms are SA and JA. SA-signaling is commonly reported as important against biotrophic pathogens and is often associated with the induction of ROS (Herrera-Vasquez et al., 2015). There are numerous reports of SA biosynthesis increase and plant defense under UV-C and/or UV-B stress, in *Arabidopsis* (Yao et al., 2011; Mintoff et al., 2015), pepper (Mahdavian et al., 2008), tobacco (Yalpani et al., 1994; Yao et al., 2011), barley (Bandurska and Cieslak, 2013) and wheat (Kovacs et al., 2014) roots and leaves. In tobacco, SA increase by supplemental UV-B is probably owing to an increase in phenylalanine ammonia lyase with subsequent accumulation of downstream PATHOGENESIS RELATED PROTEINS, e.g., PR-1, PR-3, and PR-5 defense proteins (Fujibe et al., 2000). Moreover in *Arabidopsis*, *Glutaredoxin C-9* and *PR-1* are both genes induced by UV-B and SA (Herrera-Vasquez et al., 2015). This response is most likely conserved in more plant species, for example, transcriptome data of UV-B exposed broccoli sprouts, showed increased expression of *Arabidopsis* homologs of *PR-1, PR-2*, and *PR-4* (Mewis et al., 2012). This strongly suggests that UV-B mediated regulation of SA may be a way to enhance plant defense. Altogether, UV-B supplementation could be very valuable in enhancing the response to biotrophic pathogens, such as powdery mildew, e.g., in cucumber and strawberry or Magnaporthe in rice. Besides SA, the role of JA is well established in the regulation of defense against necrotrophs and herbivores. Upon UV-B exposure, JA production increases in *Arabidopsis* (Mackerness et al., 1999), mung bean cultivar HUM12 (Choudhary and Agrawal, 2014), and *Nicotiana* sp. (Izaguirre et al., 2003; Dinh et al., 2013). Nonetheless, exceptions are reported, as UV-B does not stimulate JA production in tobacco but does enhance JA dependent induction of trypsin protease inhibitors and defense (Demkura et al., 2010), similar induction of trypsin protease inhibitors and defense by UV-B was reported in tomato (Stratmann et al., 2000). The UV-B dependent upregulation of JA derivatives in some species may be more than just a beneficial side effect on plant defense; for example methylJA protects barley seedlings from UV-B stress by increasing anti-oxidant and free radical scavenging capacities, suggesting that the elevation of endogenous JA by UV-B protects plants from high level UV-B stress (Fedina et al., 2009). This may be associated with the role of JA as a trigger for specialized metabolite production (Wasternack and Strnad, 2016). Although most reports focus on UV-B and its role in inducing plant defense responses in the plants, there is increasing evidence that also UV-C can trigger pre- and postharvest defense responses and that similar hormonal pathways are involved (Urban et al., 2016). Interestingly, very short (1s) repeated UV-C pulses stimulated plant defense responses and reduced symptom development in tomato, lettuce and pepper and grapevine upon inoculation with *B. cinerea*, *Phytophthora capsici* and *Plasmopara viticola*, respectively. Moreover, also systemic leaves showed increased resistance (Aarrouf and Urban, 2020). The effect of UV-A on plant immunity is largely unknown and has been mainly reported to stimulate biomass production and leaf size. UV-A radiation can also change the composition of phenolic compounds but less pronounced than UV-B (Verdaguer et al., 2017).

### Perspectives on Practical Use of UV in Plant Protection

Ultraviolet treatment of plants can lead to increased resistance to microorganisms in diverse ways. Besides the direct damaging effects of short-wave UV radiation on organism’s biomolecules, the induction of specialized plant metabolites and hormones in response to UV can also affect the plant-microbe interactions.

For direct effects on microorganisms to be effective, the radiation needs to reach the microorganism one wants to eliminate. High turbidity in liquids can render UV-C sterilization less effective (Jones et al., 2014). In contrast to UV-A and UV-B, UV-C is most potent to reduce infection pressure of plant pathogens by a process analogous to surface sterilization. Short treatments with UV-C kills microorganisms, while the plant remains unharmed. Similar to UV-C treatment, some pathogens like Cucumber powdery mildew (*Podosphaera xanthii*) are suppressed by exposing cucumber plants daily for 10 min to UV-B. Interestingly, the observed suppression effect is most significant when only UV-B is used with no background light and when UV-B is combined with red light exposure. The effect was least significant when UV-A or blue light was administered together with UV-B. Given that no additional suppression was observed when treating plants to UV-B, prior to inoculation, the observed effect is mainly directly on the pathogen, rather than induced plant resistance (Suthaparan et al., 2014). From the above we can infer that presence of UV-A prior to or during exposure, in the case of stimulation of photoprotective pigments, or during and after exposure, in the case of photoreactivation, can increase bacterial survival to UV radiation. UV-C is thus most effective if no UV-A (or blue) is present in the radiation source of use, and a subsequent red light or dark period is included. Epiphytic bacteria are more likely to be affected by the radiation than endophytic bacteria (e.g., *P. syringae* does both), because of poor penetration of UV-C radiation in plant tissues. The practical implementations in greenhouses and in the field are still under development or refinement [Clean light, Netherlands; UV-robot project PSKW (Zant et al., 2018)]. Also for *in vitro* tissue culture, possibilities exist using UV-C to lower infection pressure in growth rooms. Use for *in vitro* control of microorganisms on the plant by UV will depend on the transparency of the material of the container in which the plants are cultivated (Rao et al., 2011).

Perhaps the most promising for future applications changing plant-microbe interactions is the modulation of UV-B in the spectrum of the plant environment. The control of UVR8 in plants, and the associated specialized metabolism, combined with detrimental effects of UV-B radiation in microorganisms could provide a part of a steering mechanism for optimal plant growth and defense, which would rely on the capability of the plant of adapting much better to UV-B than the microorganisms. With the quickly developing UV-emitting LED light market, it is ever more possible to apply UV treatments to horticultural crops for various reasons. One major advantage of using UV-B supplementation is that the “treatment” can be instantaneously terminated, in contrast to the lasting impact of chemical plant growth regulators once taken up by the plant. However, the positive effects of UV to induce plant resistance should be taken with caution since some of the induced specialized metabolites which ward off microorganisms and insects may also be undesirable for human consumption, e.g., bitter taste.

We can thus conclude that UV-A is mainly neutral or even beneficial for the microorganisms, including the colonization of plants by plant pathogens, for DNA repair and processes associated with efficient infection. By contrast, UV-B and UV-C exposure leads to detrimental effects, often resulting in the death of the microbe and thus interesting for application in horticulture, provided the plant or beneficial organisms are damaged less by UV than its pathogen. Besides these detrimental effects on microorganisms, the upregulation of specialized metabolites in plants and microorganisms can affect the plant-microbe interactions in various ways. The list of advantages versus disadvantages on the use of different UV treatments in horticulture needs to be evaluated depending on a species and purpose-specific basis.

## Author Contributions

FV and LV conceived the project. All authors wrote the article and contributed to scientific discussion.

## Conflict of Interest

The authors declare that the research was conducted in the absence of any commercial or financial relationships that could be construed as a potential conflict of interest. The handling editor declared a past co-authorship with one of the authors FV.
