# Supplementary material for: Ultraviolet Radiation From a Plant Perspective: The Plant-Microorganism Context
Source: Front Plant Sci. 2020 Dec 15;11:597642. doi: 10.3389/fpls.2020.597642 (PMC7769811; doi:10.3389/fpls.2020.597642)
Supplement: Supplementary file 1 [file Table_1.DOCX]

**Supplementary figures and tables:**


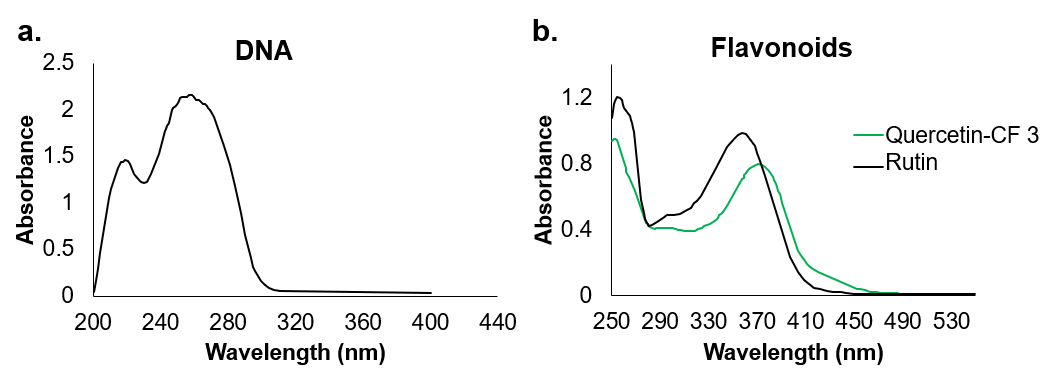


**Supplementary fig. 1: a,** Absorption spectrum of DNA. **b,** Absorption spectrum of the flavonoids quercetin-CF 3 (solid line) and rutin (dashed line) in methanol ([Tempesti et al., 2012](#_ENREF_205)).

**Supplementary table 1. Fungal diseases mentioned throughout the manuscript.** The pathogen, plant-microbe relation and host are indicated.

| Fungal pathogen | Plant-microbe relation | Main host |
| --- | --- | --- |
| *Cercospora zeae maydis* | Grey leaf spot | Maize |
| *Neurospora crassa* | Saprophyte | Scots pine |
| *Sclerotinia sclerotiorum* | White mold | Various |
| *Fusarium fujikuroi* | Bakanae disease | Rice |
| *Trichoderma atroviride* | Root endophyte | Various |
| *Aspergillus nidulans* | Saprophyte | Various |
| *Colletotrichum* | Anthracnose disease | Various |
| *Venturia inaequalis* | Apple scab disease | Woody hosts |
| *Magnaporthe grisea* | Rice blast | Rice |
| *Diplocarpon rosae* | Rose black spot disease | The genus Rosa |
| *Botrytis cinerea* | Grey mold | Various |
